# Bilateral simultaneous asymmetric hip fracture without major trauma in an elderly patient: a case report

**DOI:** 10.1186/s13256-022-03494-5

**Published:** 2022-07-16

**Authors:** Yasutaka Takagi, Hiroshi Yamada, Hidehumi Ebara, Hiroyuki Hayashi, Hiroyuki Inatani, Kazu Toyooka, Musashi Ima, Yoshiyuki Kitano, Yasuji Ryu, Aki Nakanami, Tetsutaro Yahata, Hiroyuki Tsuchiya

**Affiliations:** 1 Department of Orthopaedic Surgery, Tonami General Hospital, 1-61 Shintomi-cho, Tonami, Toyama 939-1395 Japan; 2 Department of Radiology, Tonami General Hospital, 1-61 Shintomi-cho, Tonami, Toyama 939-1395 Japan; 3 Department of Rehabilitation Medicine, Tonami General Hospital, 1-61 Shintomi-cho, Tonami, Toyama 939-1395 Japan; 4 Department of Rehabilitation Medicine, Kanazawa University Hospital, 13-1 Takara-machi, Kanazawa, Ishikawa 920-8641 Japan; 5 Department of Orthopaedic Surgery, Graduate School of Medicine, Kanazawa University, 13-1 Takara-machi, Kanazawa, Ishikawa 920-8641 Japan

**Keywords:** Hip fracture, Femoral neck fracture, Trochanteric fracture, Greater trochanteric fracture, Simultaneous, Asymmetric, Intramedullary nail, Hemiarthroplasty

## Abstract

**Background:**

Simultaneous bilateral hip fractures without major trauma in the elderly are rare and usually symmetrical. To the best of our knowledge, only two cases of bilateral simultaneous asymmetric hip fracture in the elderly without major trauma have been reported.

**Case presentation:**

We present the case of a 90-year-old Japanese man with simultaneous bilateral asymmetric hip fractures with trochanteric fracture on the right side and greater trochanteric fracture on the left side. He complained of dyspnea at midnight and was referred to our emergency department. He was admitted to the internal medicine department for bacterial pneumonia treatment. On the 8th day of hospitalization, he was referred to our orthopedic surgery department for hip pain and was found to have fractures of both hips. Computed tomography findings showed that the left femoral neck fracture was an old fracture, while the left greater trochanteric fracture and the right trochanteric fracture were fresh fractures. He was surgically treated through open reduction and internal fixation with an intramedullary nail on the right and hemiarthroplasty on the left in supine position, performed during the same surgical sessions on the 12th day of hospitalization.

**Conclusions:**

We report a new form of simultaneous bilateral asymmetric hip fracture in the elderly. The fracture types of the case were femoral trochanteric fracture and greater trochanteric fracture of the femur, which were different from the fracture types in the previously reported two cases. Clinicians should be aware of the possibility of simultaneous bilateral hip fractures, especially in the elderly.

## Background

Simultaneous bilateral hip fractures are usually associated with high-energy trauma [1], seizures [2], metabolic disorders [3], and the use of bisphosphonates [4]. They are rarely observed in the elderly after simple trauma and usually have the same fracture pattern (symmetric) [5, 6].

To the best of our knowledge, only two cases of bilateral simultaneous asymmetric hip fracture in the elderly without major trauma have been reported [7, 8]. We present a case of simultaneous bilateral asymmetric hip fracture in an elderly patient without major trauma and discuss the mechanism of injury.

## Case presentation

**Fig. 1:**
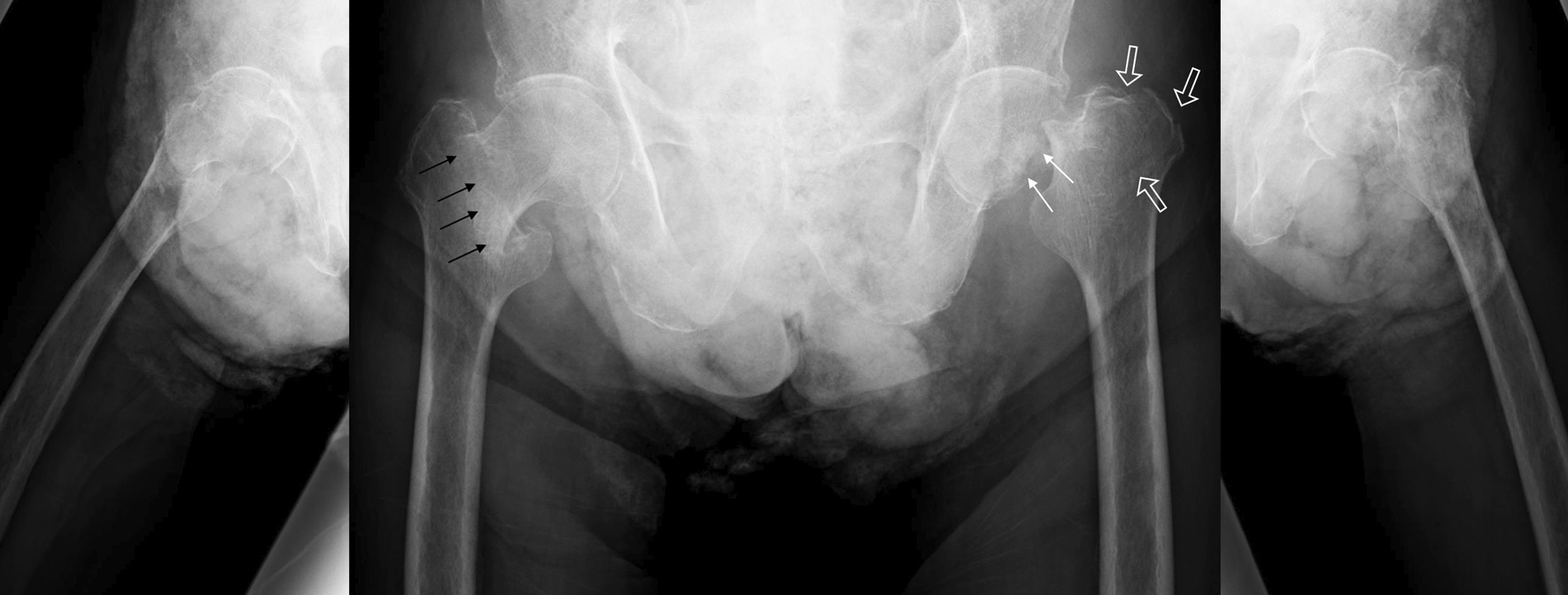
Radiography findings, showing left femoral neck fracture (white arrow), left greater trochanteric fracture (white outline arrow), and right femoral trochanteric fracture (black arrow)

We present the case of a 90-year-old Japanese man with simultaneous bilateral asymmetric hip fracture. As per his medical history, the patient had chronic obstructive pulmonary disease (COPD) and was hospitalized in the internal medicine department, had dementia and was admitted to the psychiatric department, and had cerebral contusion and symptomatic epilepsy and was hospitalized for neurosurgery. Before admission, he was able to walk with a walker. He complained of dyspnea at midnight and, therefore, visited a nearby doctor. Acute exacerbation of COPD was suspected, and he was referred to the emergency department of our institution. After admission, he was unable to walk. On the eighth day of hospitalization, he was referred to our orthopedic surgery department for hip pain and was found to have fractures of both hips. X-ray findings showed left femoral neck fracture, left greater trochanteric fracture, and right femoral trochanteric fracture (Fig. 1).

**Fig. 2:**
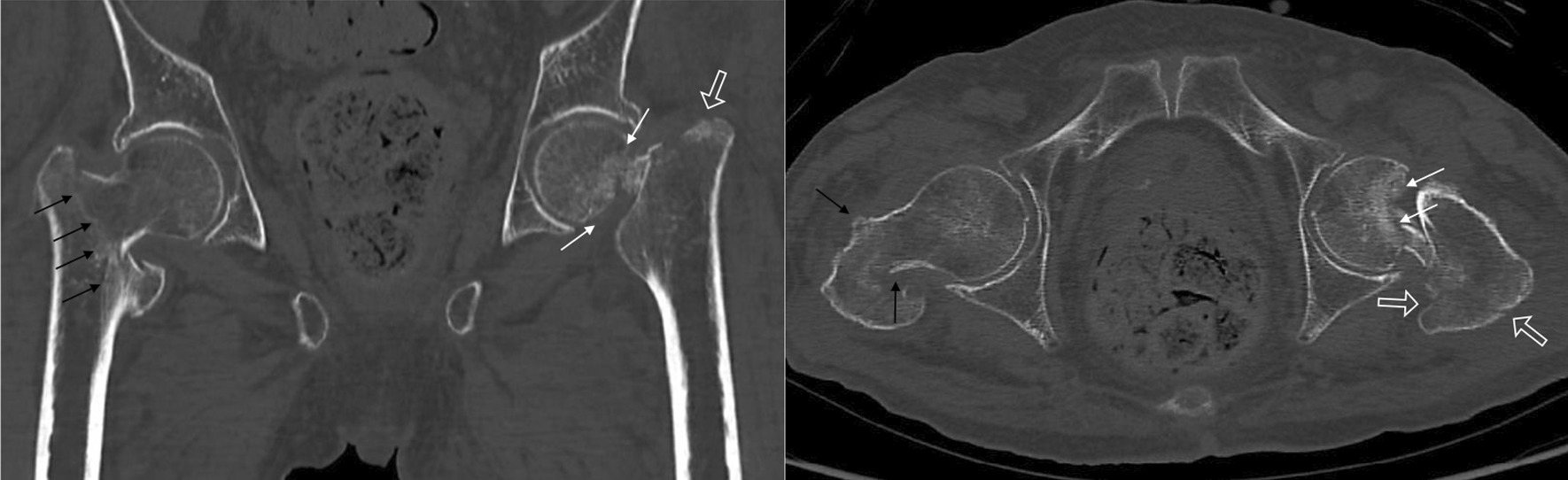
Computed tomography (CT) results showing bone osteosclerosis at the margin of the femoral head (white arrow), indicating that the left femoral neck fracture was an old fracture, while the left greater trochanteric fracture (white outline arrow) and right trochanteric fracture (black arrow) were fresh fractures

Computed tomography (CT) results showed bone osteosclerosis at the margin of the femoral head indicating that the left femoral neck fracture was an old fracture, while the left greater trochanteric and right trochanteric fractures were fresh fractures (Fig. 2).

**Fig. 3:**
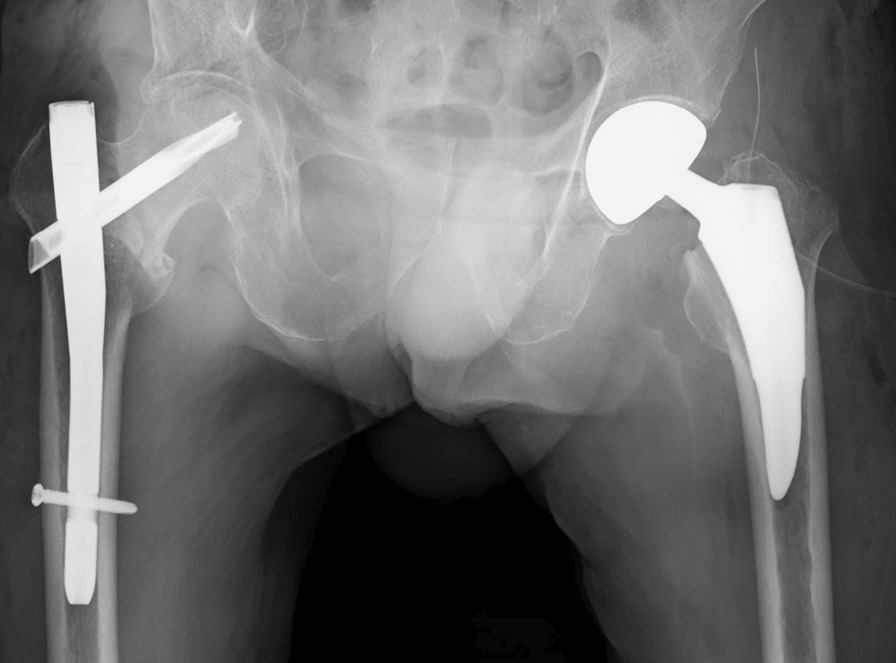
Surgical treatment. First, open reduction and internal fixation (ORIF) of the right femur with an intramedullary nail was performed. Cementless hemiarthroplasty was carried out through direct anterior approach in supine position

**Fig. 4:**
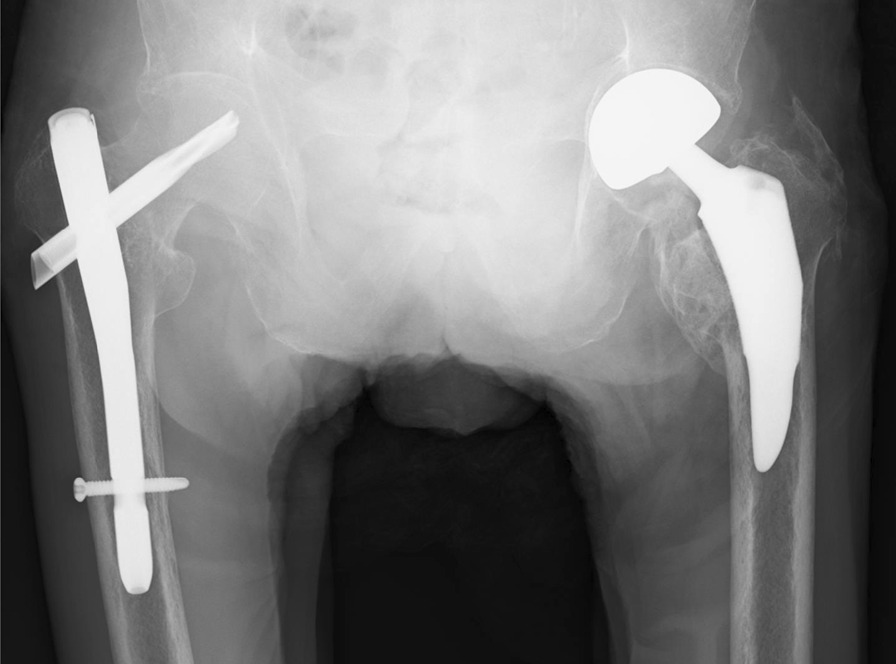
Radiography findings 13 months after the operation. Radiograph revealed absence of loosening of the left hemiarthroplasty and heterotopic ossification of the left hip joint, and bone fusion was obtained in the right trochanteric fracture

He was surgically treated through open reduction and internal fixation (ORIF) with an intramedullary nail on the right and hemiarthroplasty on the left performed during the same surgical sessions on the 12th day of hospitalization. First, ORIF of the right femur with an intramedullary nail on a traction table was performed. The first surgical operation time was 44 min. Then, the patient was moved from the traction bed to a regular surgical bed, and left hemiarthroplasty was performed for the left femoral neck fracture in supine position. A cementless hemiarthroplasty was carried out through the direct anterior approach (Fig. 3). Intraoperative findings showed absence of hematoma in the left hip joint, suggesting an old femoral neck fracture. The second surgical operation time was 1 hour 41 minutes. The preoperative hemoglobin level was 10.5 g/dL, postoperative hemoglobin level was 9.1 g/dL, and intraoperative bleeding was 200 mL; therefore, blood transfusion was not performed. The patient started physical therapy on the first postoperative day. He was able to transfer to a wheelchair and was discharged 41 days after admission. Thirteen months after the operation, he visited our outpatient clinic and X-ray examination revealed no loosening of the left hemiarthroplasty and heterotopic ossification of the left hip joint; bone fusion was observed in the right trochanteric fracture (Fig. 4).

## Discussion

Hip fractures result in a critical public health burden due to their high incidence, morbidity, mortality, and treatment cost. In a large epidemiological study, the incidence of intracapsular and extracapsular fractures were similar [5]. Hip fractures can often be bilateral, but occur on two different occasions with the same fracture pattern [6]. This phenomenon may be because each patient possesses his or her own gait and bone architecture, which could result in the same type of fall, thus causing the same anatomic type of fracture [6].

On the contrary, bilateral simultaneous hip fractures are rare and often occur in young patients due to high-energy trauma [1], seizures [2], and metabolic diseases [3]. These fractures have also been reported in patients treated with bisphosphonates and are usually atypical subtrochanteric fractures [4].

Bilateral simultaneous hip fractures are even less common in elderly patients without serious comorbidity when caused by minor trauma, and few cases have been reported in literature [8–16]. Almost all cases described had symmetrical fracture patterns and received the same treatment on both sides (arthroplasty for femoral neck fractures and ORIF with intramedullary nail for trochanteric fractures) [8–11, 13–16]. To the best of our knowledge, only two cases of bilateral simultaneous asymmetric hip fractures in the elderly after minimal trauma have been reported, one with a femoral neck fracture and the other with a femoral trochanteric fracture [7, 8].

In our case, CT results showed bone osteosclerosis at the margin of the femoral head, indicating that the left femoral neck fracture was an old fracture, while the left greater trochanteric and right trochanteric fractures were fresh fractures. Intraoperative findings showed the absence of hematoma in the left hip joint, suggesting an old femoral neck fracture. Before admission, the patient was able to walk with a walker and was determined to have had a left femoral neck fracture without displacement. After admission, he was unable to walk. A possible cause of injury is that the left femoral neck fracture was displaced after minimal trauma, resulting in simultaneous damage to the right and left trochanter. It was determined that the detection of bilateral proximal femoral fractures as a cause of difficulty in walking was delayed due to bacterial pneumonia and dementia. The injury mechanism of the greater trochanteric fracture of the left femur was diagnosed as the fact that the left lower limb was forced to adduct after minimal trauma, increasing the tension of the gluteus medius tendon.

In the first and second cases, surgery was performed in lateral decubitus position for femoral neck fracture, and changing the patient’s position during surgery was necessary [7, 8]. In our case, surgery for bilateral femoral fractures was performed in supine position for the first time, wherein it was necessary to change the patient’s position during surgery.

First, we decided to treat the trochanteric fracture, which was performed on a traction table and stabilized with an intramedullary nail after reduction. Then, the patient was moved from the traction bed to the regular surgical bed. Left hemiarthroplasty was performed on the contralateral side in supine position. We opted for this sequence for two reasons: first, to easily switch the patient from the traction table to the regular surgical bed and vice versa, and second, to avoid the risk of dislocating the prosthesis when moving the patient to the traction table.

To the best of our knowledge, bilateral simultaneous asymmetric hip fractures with femoral trochanteric fractures and greater trochanteric fractures has not been previously reported in literature. The fracture types in this case were femoral trochanteric fracture and greater trochanteric fracture of the femur. This is a new form of simultaneous bilateral asymmetric hip fractures. Clinicians should be aware of the possibility of simultaneous bilateral hip fractures, especially in the elderly.

## Conclusion

We report a new form of simultaneous bilateral asymmetric hip fractures in the elderly. The fracture types reported were femoral trochanteric fracture and greater trochanteric fracture of the femur, which were different from the fracture types of the previous two cases. Clinicians should be aware of the possibility of simultaneous bilateral hip fractures, especially in the elderly.

## Data Availability

Medical imaging data will not be shared because it is not fully anonymous.

## Abbreviations

COPD: Chronic obstructive pulmonary disease
CT: Computed tomography
ORIF: Open reduction and internal fixation
